# Non-Coding RNAs and Extracellular Vehicles: Their Role in the Pathogenesis of Gestational Diabetes Mellitus

**DOI:** 10.3389/fendo.2021.664287

**Published:** 2021-05-20

**Authors:** Tie-Ning Zhang, Wei Wang, Xin-Mei Huang, Shan-Yan Gao

**Affiliations:** ^1^ Department of Clinical Epidemiology, Shengjing Hospital of China Medical University, Shenyang, China; ^2^ Clinical Research Center, Shengjing Hospital of China Medical University, Shenyang, China; ^3^ Department of Pediatrics, Shengjing Hospital of China Medical University, Shenyang, China; ^4^ Department of Endocrinology, Shanghai Fifth People’s Hospital, Fudan University, Shanghai, China

**Keywords:** lncRNA, miRNA, circRNA, extracellular vehicles, GDM

## Abstract

Gestational diabetes mellitus (GDM) is defined as glucose intolerance with onset or first recognition in the second or third trimester of pregnancy. GDM has a considerable impact on health outcomes of the mother and offspring during pregnancy, delivery, and beyond. Although the exact mechanism regarding GDM remains unclear, numerous studies have suggested that non-coding RNAs, including long non-coding (lnc)RNAs, microRNAs, and circular RNAs, were involved in the pathogenesis of GDM in which they played vital regulatory roles. Additionally, several studies have revealed that extracellular vehicles also participated in the pathogenesis of GDM, highlighting their important role in this disease. Considering the lack of effective biomarkers for the early identification of and specific treatment for GDM, non-coding RNAs and extracellular vehicles may be promising biomarkers and even targets for GDM therapies. This review provides an update on our understanding of the role of non-coding RNAs and extracellular vehicles in GDM. As our understanding of the function of lncRNAs and extracellular vehicles improves, the future appears promising for their use as potential biomarkers and treatment targets for GDM in clinical practice.

## Introduction

Gestational diabetes mellitus (GDM), defined as any degree of glucose intolerance with onset or first recognition during pregnancy, is now one of the most common complications of pregnancy affecting up to 9–26% of the obstetric population (1, 2). The prevalence of GDM has been rising substantially over recent decades and is expected to continue, along with an increase in the prevalence of maternal obesity. In 2017, it was estimated that one in every seven live births globally were affected by GDM (3). GDM has a considerable impact on health outcomes of the mother and offspring during pregnancy, delivery, and beyond (4). For mothers, GDM is an established risk factor for developing type 2 diabetes (T2DM) in later life (5). For offspring, the adverse intrauterine environment causes epigenetic changes in the fetus that may contribute to the development of metabolic disorders including childhood obesity (6), T2DM, and metabolic syndrome (7), the so-called vicious cycle of diabetes (8). These potential adverse outcomes in both mother and offspring underpin the importance of correctly diagnosing and managing GDM. Novel diagnostic markers [such as non-coding RNAs and extracellular vehicles (EVs)] linked to insulin resistance and β-cell dysfunction to identify women at high risk for the development of GDM are useful to target therapy and potentially prevent its development. Evidence from well-powered, prospective observational and interventional studies indicates a role for diet and prenatal exercise in the development of GDM (9, 10). Compelling data suggest that genetic factors also play a role in GDM (11). The contribution of these modifiable lifestyle and genetic factors highlights the potential for complex mechanistic pathways underlying GDM. However, a complete understanding of the exact molecular mechanisms involved in GDM requires further research.

Non-coding RNAs are a class of RNA that are mainly responsible for the regulation of many cell signaling pathways. These molecules can be divided into long non-coding RNAs (lncRNAs), microRNAs (miRNAs), and circular RNAs (circRNAs). With the development of chip array and next-generation high-throughput sequencing (NGS), recent studies highlighted the critical role of non-coding RNAs in different types of diseases and how they were involved in several biological processes (12, 13). Notably, the expression patterns of lncRNAs, miRNAs, and circRNAs in GDM were found to differ according to several clinical and experimental studies. Further bioinformatics analyses revealed that non-coding RNAs function in different types of biological processes, suggesting a potential role for non-coding RNAs in GDM. Except for non-coding RNAs, EVs are reported to be active in cell-to-cell communication, both in normal tissues and in diseased conditions such as cancer, due to the presence of functional proteins and also non-coding RNA (14, 15). Although the exact role of EVs in GDM remains unclear, published studies have shown that EVs from different tissues in the body might have vital roles in GDM, suggesting EVs could participate in its pathogenesis. A firm understanding of the regulatory and functional role of non-coding RNAs as well as EVs may be key to inducing alterations in GDM. Substantial studies have explored the regulatory process from the perspective of non-coding RNAs and EVs in an effort to provide new insights into the regulatory mechanisms of GDM.

To enhance our understanding of non-coding RNAs and EVs in GDM, a comprehensive summary of their roles in this disease is needed. Herein, this review will discuss how non-coding RNAs and EVs affect the pathogenesis of GDM and their potential use as biomarkers or therapeutic targets for this disorder.

## Biogenesis of Non-Coding RNAs and EVs

### Biogenesis of lncRNAs

**Figure 1:**
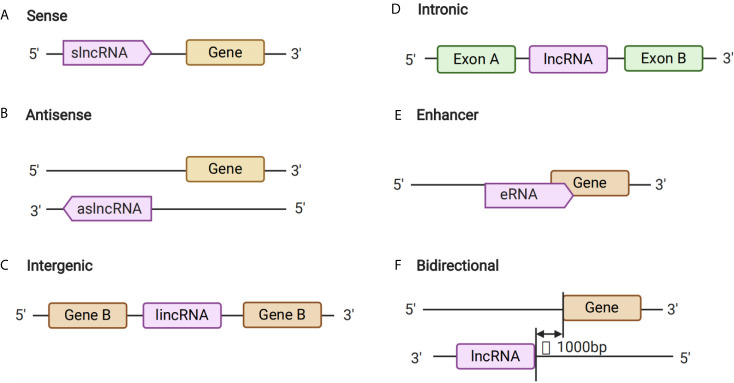
Long Non-coding RNA (lncRNAs) Biogenesis. **(A–F)** The different kinds of biogenesis of lncRNAs.

Long non-coding RNAs are a type of non-coding RNA with a length that exceeds 200 nt. Such lncRNAs contribute to transcriptional and post-transcriptional functions, and can be broadly classified either as signaling, decoy, guide, or scaffold molecules according to their functions (16). Notably, although the primary sequence of lncRNAs is poorly conserved, it can be partially compensated for through a high degree of structural conservation (17). Previous studies have shown that lncRNAs can be transcribed from conserved genomic regions (16) and back-splicing of exons, which could form circRNAs and also generate lncRNAs (18, 19) (**Figure 1**). Long non-coding RNAs have roles in several important functions in cells, including chromatin rearrangement, histone modification, modification of alternative splicing genes, as well as the regulation of gene expression, and thus mediate different types of biological processes. Therefore, lncRNAs might play important roles in the pathogenesis of various diseases, including GDM.

### Biogenesis of miRNAs

**Figure 2:**
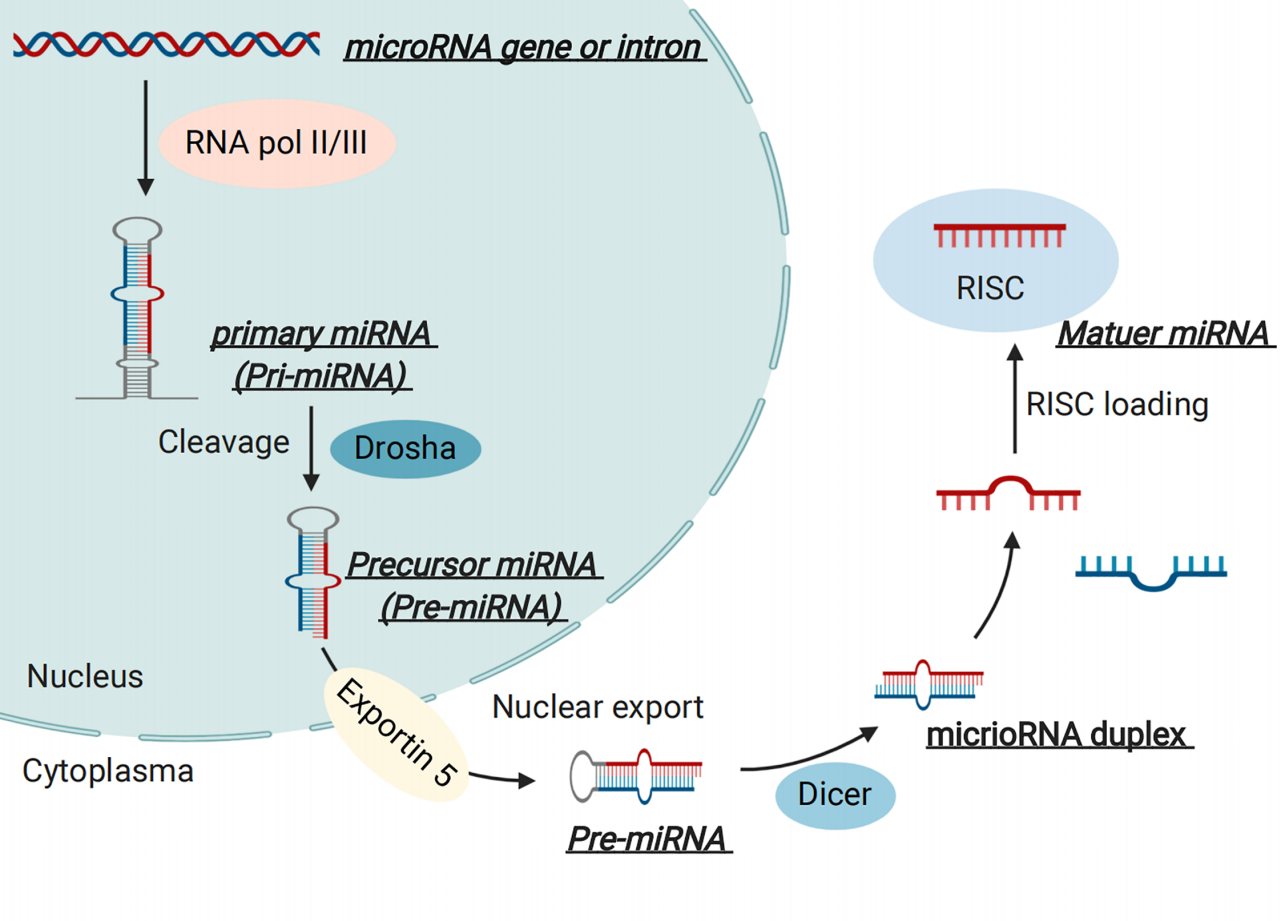
MicroRNA (miRNA) Biogenesis.

MicroRNAs are endogenous non-coding RNA molecules with lengths of 19–22 nt (20). The biogenesis of miRNAs has been described in our previous review (21). Briefly, miRNAs are mainly transcribed by RNA polymerase II and thus result in a primary miRNA (called pri-miRNA), approximately 500–3000 nucleotides in length (22). The pri-miRNA is then cleaved into a premature miRNA (called pre-miRNA), about 70-80 nucleotides in length, by a “microprocessor complex” (23). Furthermore, pre-miRNA is then exported into the cytoplasm with the help of the nuclear export transporter, exportin 5, which processes about a 22 nucleotide “miRNA duplex” by interacting with RNase III endonuclease Dicer protein and a co-factor double-stranded transactivation-responsive RNA binding protein (24). The miRNA duplex is integrated into an “RNA-induced silencing complex” after binding to an Argonaute and glycine tryptophan repeat-containing protein, where they bind to partial or full-complementary sequences in the 3’ untranslated region (3’-UTR) or 5’-UTR of the target mRNA, and thus participate in the regulatory process of gene expression (25, 26)(**Figure 2**). Notably, numerous studies have suggested critical roles for miRNAs in the pathogenesis of GDM based on clinical and experimental research.

### Biogenesis of circRNAs

**Figure 3:**
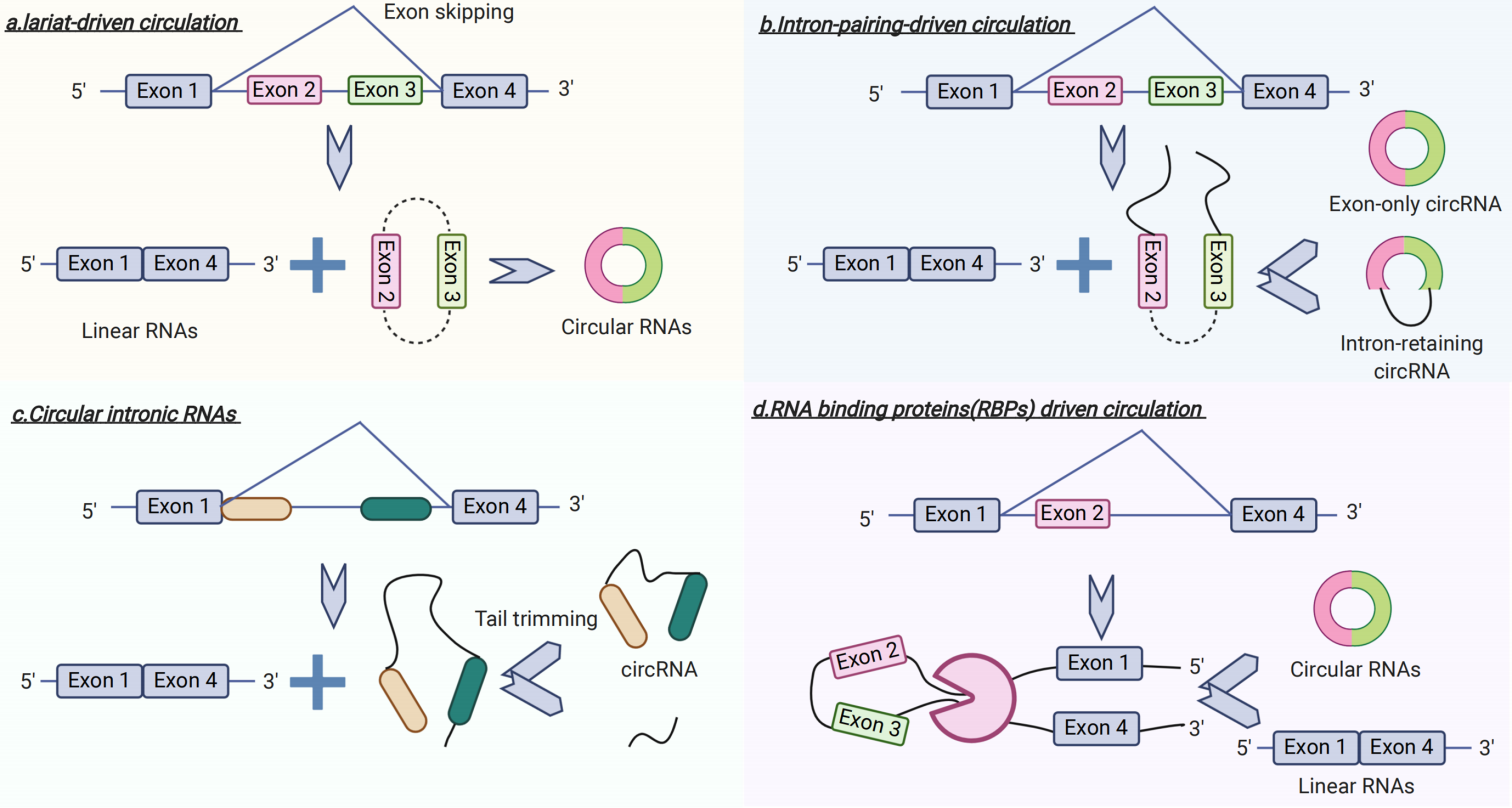
Circular RNA (circRNA) Biogenesis.

Recently, circRNAs have become a focus of research in the field of non-coding RNAs (27, 28). In contrast to linear RNA, circRNA forms a covalent, closed-loop structure lacking both 5’-3’ polarity by exon or intron circularization and a poly-A tail (28) (**Figure 3**). Circular RNAs mainly come from the exons of protein-coding genes and are not formed by the normal model of RNA splicing (29). A prior study has shown that circRNAs are formed *via* two different mechanisms of exon circularization, lariat-driven circularization, and intron-pairing–driven circularization (18). In addition, with regard to introns between exons, when these form a circular structure, they are removed or retained to form an exon-only circRNA or intron retaining circRNA called EIciRNA (18, 30). What is more, circRNAs can also be generated from the circularization of two flanking intronic sequences (31, 32). To date, several studies have indicated that circRNAs have potential roles in the pathogenesis of GDM.

### Biogenesis of EVs

**Figure 4:**
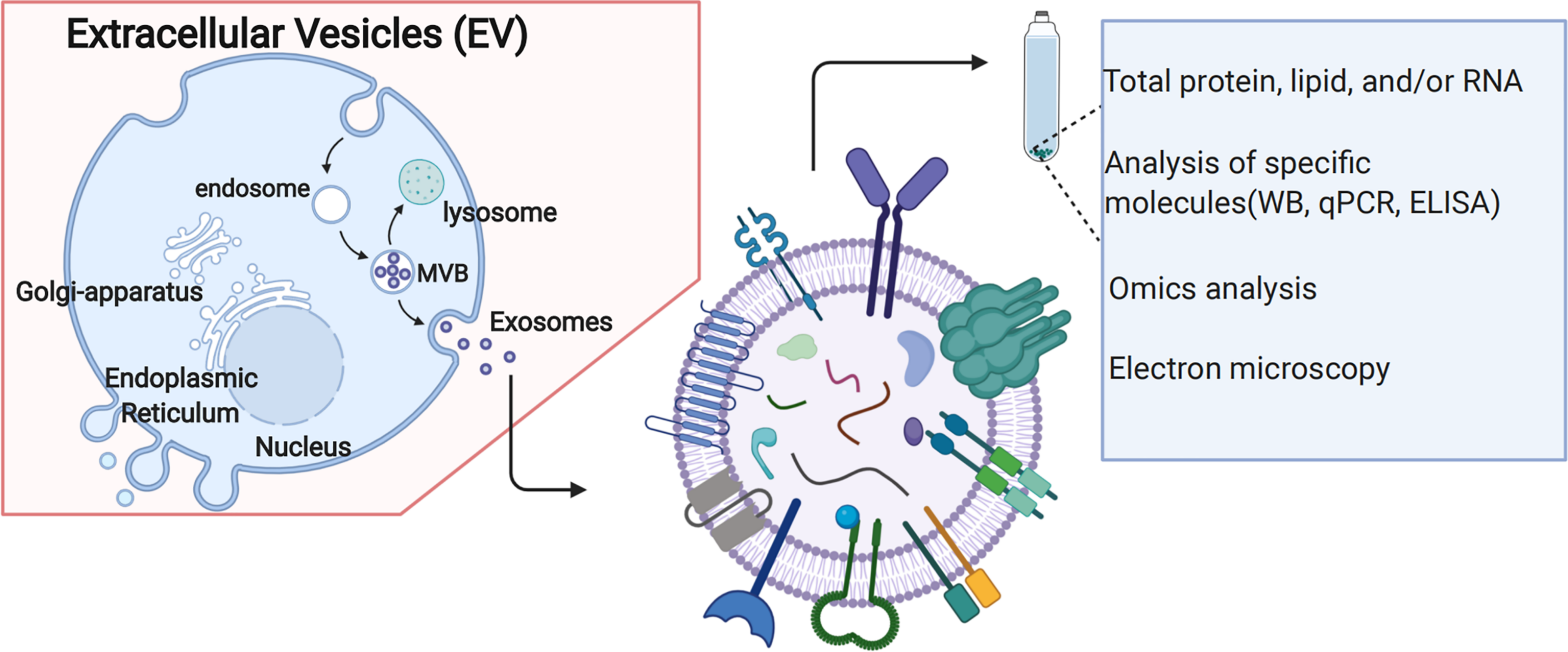
Extracellular vehicles (EVs) Biogenesis.

The biogenesis of EVs is shown in **Figure 4**. It has often been described as an endosomal sorting complex required for transport (ESCRT)-dependent or ESCRT-independent mechanisms (33). ESCRT consists of four different protein complexes: ESCRT-0, -I, -II, and -III, and an associated AAA ATPase Vps4 complex (34). As for ESCRT-dependent processes, EVs biogenesis starts within the endosomal system; early endosomes mature into late endosomes or multivesicular bodies (MVBs). During this process, the endosomal membrane invaginates to generate intraluminal vesicles (ILVs) in the lumen of the organelles. Notably, ESCRT machinery is important in this process (35). In addition, several studies suggest that MVB biogenesis can occur without ESCRTs. For example, it has been shown that despite the simultaneous silencing of key subunits of all four ESCRT complexes, ILVs are still formed in MVBs, thus highlighting the presence of ESCRT-independent mechanisms (36). The study showed that several important proteins, such as tetraspanins, transmembrane proteins enriched in EVs, are involved in ESCRT-independent EVs release in which they have a key role (37). In addition to proteins, lipids are also essential players in vesicular transport (38); both types of molecules collaborate closely in essential processes intrinsic to vesicular transport, such as membrane deformation, fission and fusion (39). Notably, ESCRT -dependent or ESCRT-independent mechanisms might not be entirely separated (40). These two pathways might work synergistically, and different subpopulations of EVs likely depend on different machineries. Recent studies have also highlighted the potential role of EVs in the pathogenesis of GDM, suggesting using EVs as new biomarkers or therapeutic targets.

## Role of Non-Coding RNAs and EVs in GDM

Non-coding RNAs and EVs are linked and also appear to vary during the pathogenesis of GDM. Non-coding RNAs and extracellular vehicles have important regulatory roles in mediating different kinds of biological processes and phenotypes, such as cell apoptosis, cell proliferation, and so on, and thus contribute to pathology of GDM. Up to now, a majority of studies have concentrated on the relationship between miRNAs and GDM, while a relatively small number of studies have explored the relationship between circRNAs/lncRNAs/EVs in the pathogenesis of GDM.

### Role of lncRNAs in GDM

Recently, NGS has become a powerful method to detect differing expressions of lncRNAs. Tang et al. performed NGS using placental samples from GDM patients and healthy controls: It was reported that 86 lncRNAs were up-regulated while 86 lncRNAs were down-regulated, suggesting different expression patterns and potential roles for lncRNAs in GDM (41). Compared with placental tissue, the study conducted by Lu et al. described 197 increased lncRNAs in peripheral blood samples from GDM patients compared with pregnant controls based on lncRNA array analyses (42). Except for patients’ specimens, another study based on a mouse GDM model found that 52 lncRNAs and 82 mRNAs were differentially expressed in the placentas of mice fed a high-fat diet during pregnancy (as a GDM model); 120 lncRNAs as well as 202 mRNAs were differentially expressed in gonadal fat tissue through NGS analyses (43). In addition, prior studies showed that genes, miRNAs, and their shared target lncRNAs can form lncRNA-associated feed-forward loops (lnc-FFLs), in which genes and miRNAs co-ordinate to regulate lncRNA expression (44). Notably, lnc-FFLs participate in many biological processes, such as cell development and differentiation, and are related to different types of diseases (45, 46). Also, lnc-FFLs have a role in GDM. For example, Fu et al. constructed a global lnc-FFL network regarding GDM. They found strong associations between dysregulated glycometabolism- and hormone-related lnc-FFLs in GDM (47). Additionally, they discovered that dysregulated lnc-FFLs were enriched in the thyroid hormone signaling pathway, and that several drug-repurposing candidates (including hormonal drugs) could be identified based on lnc-FFLs in GDM (47), suggesting different expression patterns in this disease. Therefore, lncRNAs might be essential to the pathogenesis of GDM.

Recent studies have found that several specific lncRNAs were up-regulated in GDM and participated in regulatory processes (**Table 1**). For instance, lncRNA MALAT1 expression was higher in placental tissues from a GDM group compared to a healthy pregnant women group (57). At a molecular level, the downregulation of lncRNA MALAT1 might inhibit the secretion of inflammatory factors and suppress the proliferation, invasion, and migration of GDM placental trophoblastic cells, possibly mediated by transforming growth factor (TGF)-β and NF-κB signaling pathways (57). In addition, compared to pregnant women without GDM, lncRNA MEG3 levels were also significantly elevated in the blood and placental villous tissues of those with GDM. Knockdown of lncRNA MEG3 significantly enhanced HTR-8/SVneo cell viability, promoted cell migration/invasion, and reduced cell apoptosis (49). Similarly, lncRNA MEG3 was overexpressed in human umbilical vein endothelial cells (HUVECs) from GDM patients compared with healthy controls, which might have led to the downregulation of miR-370-3p and upregulation of AFF1, mainly through inhibiting the phosphatidylinositide 3-kinase (PI3K)/AKT pathway at a molecular level and thus influencing the process of GDM (50).

**Table 1:** Selected lncRNAs and circRNA involved in GDM.

lncRNAs	Expressionin GDM	Target Gene	Key Points of Investigation	Model (*In Vitro, In Vivo*, Human)	Sample Type	Sample Size	Ref.
lncRNA MALAT1	Up	–	Knockdown of lncRNA MALAT1 reduced the expression of TGF-beta and NF-kappa B	Human, *in vitro*	Placental tissues	78 GDM vs 38 controls	(48)
lncRNA MEG3	Up	miR-345-3p	lncRNA MEG3 overexpression significantly inhibited HTR-8/SVneo cell viability, and prevented cell migration and invasion in addition to inducing cell apoptosis	Human, *in vitro*	Placental tissues and peripheral blood	20 GDM vs 20 controls	(49)
lncRNA MEG3	Up	miR-370-3p	MEG3 overexpression leads to downregulated miR-370-3p and upregulates AFF1 mainly through inhibiting PI3K/AKT pathway	Human, *in vitro*	HUVECs from pregnancy	16 GDM vs 18 controls	(50)
lncRNA H19	Down	–	H19 affects insulin secretion by altering the cellular function of islet cells	In vivo	–	–	(51)
lncRNA DANCR	Down	miR-33a-5p	lnc-DANCR-miR-33a-5p-ABCA1 signaling cascade plays a crucial role in GDM	Human, *in vitro*	Peripheral blood	12 GDM vs 12 controls	(52)
lncRNA PVT1	Down	–	105 differentially expressed genes after PVT1 knockdown	*in vitro*	–	–	(53)
Hsa_circRNA_0054633	Up	–	Hsa_circ_0054633 showed a significant diagnostic value	Human	Neonatal cord blood, placental tissues and peripheral blood	40 GDM vs 40 controls (2nd trimester), 65 GDM vs 65 controls (third trimester)	(54)
Hsa_circRNA_0005243	Down	–	Knockdown of hsa_circ_0005243 reduced the expression of β-catenin and increased nuclear NF-κB p65 nuclear translocation	Human, *in vitro*	Placental tissues and peripheral blood	20 GDM vs 20 controls	(55)
Hsa_circRNA_102893	Down	–	Hsa_circ_102893 showed a significant diagnostic value	Human	Peripheral blood	12 GDM vs 12 controls (training set), 18 GDM vs 18 controls (test set)	(56)

circRNA, circular RNA; lncRNA, long non-coding RNA; miRNA, microRNA; GDM, Gestational diabetes mellitus.

In addition to a high expression level, lncRNA was also found to be down-regulated in GDM and to play a key role in regulatory processes. H19 is the first lncRNA reported to be associated with GDM, which affected insulin secretion by altering the cellular function of islet cells (51). Notably, further study showed that H19 participated in alterations in DNA methylation in umbilical cord blood exposed to intrauterine hyperglycemia. Such alterations had a functional role in regulating genes associated with insulin-like growth factor (IGF)2, providing evidence of strong associations between H19 and methylation in GDM (58). In addition, lnc-DANCR functioned as a sponge for miR-33a-5p and thus to antagonize the function of miR-33a-5p, which was upregulated in blood samples from GDM and contributed to cell growth and insulin production by targeting ABCA1 (52). Moreover, the expression level of lncRNA PVT1 was lower in placentas from GDM patients than in healthy placentas, which might disrupt the function of trophoblast cells through the PI3K/AKT pathway (53). However, considering various lncRNAs differed in their expression based on the results of RNA- sequencing (RNA-seq) or chip array, the role of lncRNAs in the pathogenesis of GDM has not been fully elucidated. Future research is required to investigate how lncRNAs influence GDM and to determine the exact molecular signaling pathways involved.

### Role of miRNAs in GDM

Compared with lncRNAs, a large number of studies have demonstrated that miRNAs play critical roles in the pathogenesis of GDM (48, 59–62). Based on NGS, the expression patterns of miRNAs in GDM differed. For example, although the sample number was relatively small, the study conducted by Tang et al. found that only two miRNAs were up-regulated, while two miRNAs were down-regulated in a GDM group (n=3) compared with healthy controls (n=3) based on NGS using placental samples (41). As for peripheral blood samples from GDM, the study performed by Zamanian et al. identified 12 differentially expressed miRNAs that were mostly up-regulated (63). Similarly, the study conducted by Zhu et al. reported 32 miRNAs that were differentially expressed in GDM, including 12 miRNAs that were upregulated and 20 that were downregulated in blood samples based on RNA-seq (64). However, further research on a large scale is still needed in order to provide a comprehensive understanding of the expression patterns of miRNAs in GDM based on NGS.

Several studies have shown that a few specific miRNAs were up-regulated in GDM, and thus mediated its pathogenesis (**Table 2**). For example, GDM cases showed a 4-fold increase in miRNA-19a and a 4.7-fould increase in miRNA-19b expression compared to healthy control individuals, suggesting a potential biomarker value for miR-19 (65). In addition, the expression of miR-140 was also up-regulated in GDM patients. Further experiments showed that miR-140-3p suppressed insulin receptor-α and IGF1R expression *via* targeting 3’UTRs, thus contributing to defective placental insulin receptor signaling in GDM patients (70). Moreover, miR-770-5p was up-regulated in GDM patients compared with healthy controls (78). At a molecular level, miR-770-5p negatively regulated the expression of TRIAP1 in INS-1 cells, while the inhibition of miR-770-5p enhanced INS-1 cell proliferation and suppressed cell apoptosis (78). As for miR-657, a study performed by Wang et al. suggested its up-regulation regulated interleukin (IL)-37 and the activation of NF‐κB, and also regulated macrophage proliferation, migration, and polarization by targeting FAM46C, thus participating in the pathogenesis of GDM (81, 97). Additionally, expression levels of miR-137 were increased in the placental tissues of women with severe GDM; miR-137 suppressed the viability and migration of trophoblasts *via* the downregulation of fibronectin type II domain-containing protein 5 (68). A study by Xu et al. also found that miR-503 was markedly upregulated in placental tissue from GDM patients, as was similarly found in peripheral blood specimens; the high level was positively correlated with the blood glucose concentration (79). At a molecular level, a further study suggested that miR-503 regulated functions of pancreatic β-cells by targeting the mammalian target of rapamycin (mTOR) pathway, suggesting that targeting the miR-503/mTOR axis could serve as a novel therapeutic target for GDM (79). A study performed by Zhao et al. also found that miR-518d expression was higher in placentas taken from patients with GDM compared with control placentas, which may be associated with the pathogenesis of GDM *via* an effect on the regulation of peroxisome proliferator-activate receptors (PPAR)α expression (80). Another study showed that miRNA-340 in maternal whole blood cell samples was elevated in GDM patients compared with healthy controls, and was found to be inversely regulated by glucose and insulin (76). Furthermore, miR-137 was upregulated in the plasma of GDM patients, which enhanced the interaction between endothelial cells and monocytes, suggesting a key role for miR-137 in GDM (69). In HUVECs from GDM, miR-101 was up-regulated; the inhibition of miR-101 increased the enhancer of zeste homolog 2 (EZH2) expression and improved GDM HUVEC function, suggesting GDM impairs HUVEC function *via* miR-101 upregulation (67). Notably, other studies also reported miRNAs, such as miR-195-5p (73), miR-330-3p (75), and miR-98 (66), were up-regulated and related to GDM. These findings indicate that up-regulated miRNAs are associated with the pathogenetic processes of GDM.

**Table 2:** Selected miRNAs involved in GDM.

miRNAs	Expression in GDM	Key Points of Investigation		Model (*In Vitro, In Vivo*, Human)	Sample Type	Sample Size	Ref.
miRNA-19a	Up	–		Human	Peripheral blood	100 GDM vs 100 controls	(65)
miRNA-19b	Up	–		Human	Peripheral blood	100 GDM vs 100 controls	(65)
miR-98	Up	Up-regulation of miR-98 in the placental tissues of human GDM is linked to the global DNA methylation *via* targeting Mecp2		Human, *in vitro*	Placenta	193 GDM vs 202 controls	(66)
miR-101	Up	EZH2 is both a transcriptional inhibitor and a target gene of miR-101 in HUVECs, and it contributes to some of the miR-101-induced defects of GDM-HUVECs		Human, *in vitro*	HUVECs	22 GDM vs 24 controls	(67)
miR-137	Up	Upregulating miR-137 in HTR-8/SVneo cells downregulates the expression levels of FNDC5		Human, *in vitro*	Placenta	19 GDM vs 20 controls	(68)
miR-137	Up	Upregulating miR-137 enhanced the interaction between endothelial cells and monocytes		Human, *in vitro*	Peripheral blood	11 GDM vs 12 controls	(69)
miR-140	Up	miR-140-3p suppresses IR-α and IGF1R expression *via* targeting the 3’UTRs		Human, *in vitro*	Placental tissues	33GDM vs 20 controls	(70)
miR-142-3p	Up	miR-142-3p promotes the survival of pancreatic β cells through targeting FOXO1		*In vivo* and *in vitro*	–	–	(71)
miR-145-5p	Up	Targeting APOB, IRS1, RETN, and GCG		Human	Peripheral blood	2 GDM vs 2 controls	(63)
miR-146b-3p	Up	Targeting IL-6		Human	Peripheral blood	2 GDM vs 2 controls	(63)
miR−195−5p	Up	miR-195-5p may inhibit cell viability, proliferation and promote apoptosis by targeting EZH2		*In vitro* (HUVEC cell line)	–	–	(72)
miR-195-5p	Up	miR-195-5p targeted large number of important genes regarding metabolism		Human	Peripheral blood	13 GDM vs 9 controls	(73)
miR-325	Up	Targeting GCG		Human	Peripheral blood	2 GDM vs 2 controls	(63)
miR-335-5p	Up	miR‐335‐5p can promote insulin resistance and suppress pancreatic islet β‐cell secretion in GDM *via* activating the TGF‐β signaling pathway and suppressing the expression of VASH1		*In vivo*	–	–	(74)
miR-330-3p	Up	miR-330-3p target genes analysis revealed major modulators of beta-cell proliferation and of insulin secretion, such as the experimentally validated genes E2F1 and CDC42 as well as AGT2R2		Human	Peripheral blood	21 GDM vs 10 controls	(75)
miRNA-340	Up	mRNA and protein expression of PAIP1, a miRNA-340 target gene, was found down-regulated in GDM		Human	Peripheral blood	8 GDM vs 8 controls	(76)
miR‐351	Up	miR‐351 protects against IR and liver gluconeogenesis by repressing the PI3K/AKT pathway through regulating FLOT2		*In vivo, in vitro*	–	–	(77)
miR-770-5p	Up	miR-770-5p is a vital regulator in pancreatic β-cell proliferation, apoptosis and insulin secretion by targeting TRIAP1		Human, *in vitro*	Peripheral blood	30 GDM vs 30 controls	(78)
miR-365a-3p	Up	Targeting IL-6		Human	Peripheral blood	2 GDM vs 2 controls	(63)
miR-503	Up	miR-503 regulated functions of pancreatic β-cells by targeting the mTOR pathway		Human, *in vitro*	Placental tissues and peripheral blood	3 GDM vs 3 controls (placenta); 25 GDM vs 25 controls (peripheral blood)	(79)
miR-518d	Up	Upregulation of miR-518d may be associated with the pathogenesis of GDM *via* an effect on the regulation of PPARα expression		Human, *in vitro*	Placenta	40 GDM vs 40 controls	(80)
miR-520e	Up	Targeting APOB		Human	Peripheral blood	2 GDM vs 2 controls	(63)
miR-568	Up	Targeting IL-6		Human	Peripheral blood	2 GDM vs 2 controls	(63)
miR-574-3p	Up	Targeting IL-6		Human	Peripheral blood	2 GDM vs 2 controls	(63)
miR-583	Up	Targeting ALB		Human	Peripheral blood	2 GDM vs 2 controls	(63)
miR-657	Up	Dysregulation of miR‐657 contributes to the pathogenesis of GDM *via* IL‐37/NF‐κB signaling axis		Human, *in vitro*	Placenta	48 GDM vs 46 controls	(81)
miR-20a-5p	Down	–		Human	Peripheral blood	28GDM vs 53 controls	(82)
miR-21	Down	miR-21 inhibits cell growth and infiltration by up-regulating PPAR-α		Human, *in vitro*	Placental tissues and peripheral blood	137 GDM vs 158 controls	(83)
miR-29b	Down	Downregulation of miR-29b may be related with GDM partially *via* increasing the expression of HIF3A		Human, *in vitro*	Placental tissues	204 GDM vs 202 controls	(84)
miR-29b	Down	Effect of miR-29b on GDM rats targeting PI3K/Akt signal		*In vivo* (GDM rat model)	–	–	(85)
miR-96	Down	miR-96 enhanced β-cell function, whereas PAK1 inhibited β-cell function and cell viability		Human, *in vitro*	Peripheral blood	3 GDM vs 3 controls	(86)
miR-122	Down	miR-122 levels were regulated both *in vitro* through PPARγ activation and *in vivo* through a maternal diet enriched in PPAR ligands		*In vivo* and *in vitro*	–	–	(87)
miR-138-5p	Down	Overexpression of miR-138-5p inhibits the migration and proliferation of HTR-8/SVneo *via* TBL1X		Human, *in vitro*	Placenta	8 GDM vs 8 controls	(88)
miR-143	Down	Down-regulation of miR-143 mediates the metabolic switch from oxidative phosphorylation to aerobic glycolysis in placenta of women with GDM		Human, *in vitro*	Placenta	6 GDM vs 6 controls	(89)
miR-155-5p	Down	–		Human	Peripheral blood	14 GDM vs 27 controls	(90)
miR-181a	Down	–		Human	Umbilical-cord blood cells	20 GDM vs 20 controls	(91)
miR-193b	Down	miR-193b suppresses apoptosis and autophagy *via* targeting IGFBP5		Human, *in vitro*	Peripheral blood	20 GDM vs 20 controls	(92)
miRNA-221	Down		Overexpression of miRNA-221 could stimulate insulin secretion, cell proliferation and suppress apoptosis *via* PAK1 in INS-1 cells	*In vivo* and *in vitro*	–	–	(93)
miR-345-3p	Down		Negative regulation of BAK1 by miR-345-3p	Human, *in vitro*	Placental tissues and peripheral blood	30 GDM vs 30 controls	(94)
miR-371a-5p	Down		Targeting IL-6	Human	Peripheral blood	2 GDM vs 2 controls	(63)
miR-374b-5p	Down		Targeting IL-6	Human	Peripheral blood	2 GDM vs 2 controls	(63)
miR-494	Down		miR-494 knockdown exhibited decreased insulin secretion and proliferation, as well as stimulated apoptosis in pancreatic β-cell by targeting PTEN	Human, *in vitro*	Peripheral blood	20 GDM vs 20 controls	(95)
miR-609	Down		Targeting ALB and IGF2	Human	Peripheral blood	2 GDM vs 2 controls	(63)
miR-873	Down		Downregulation of miR-873 upregulates the expression of IGFBP2 and activated PI3K/AKT/mTOR axis	*In vivo* (GDM rat model)	–	–	(96)
miR-875-5p	Down		Targeting TNF, LEP, and IRS1	Human	Peripheral blood	2 GDM vs 2 controls	(63)

miR, microRNA; GDM, Gestational diabetes mellitus.

However, substantial studies showed that several specific miRNAs were down-regulated in GDM. For instance, Li et al. showed that miR-345-3p expression was significantly decreased in placental tissues and peripheral blood of patients with GDM compared with healthy pregnant women (94). Further *in vitro* investigation showed that miR-345-3p overexpression exhibited a protective role in GDM by inhibiting HTR8-/SVneo cell apoptosis, and promoting cell proliferation and migration *via* the targeting of Bcl2 antagonistic/killer 1 (94). Similarly, miR-193b was downregulated in GDM patients, while an *in vitro* study found that its aberrantly low expression in high glucose (HG)-induced trophoblasts led to cell apoptotic events by upregulating insulin-like growth factor binding protein (IGFBP5)-induced autophagy, which might result in GDM (92). In addition, miR-21 was down-regulated in the sera and placentas of GDM patients compared to healthy pregnant women, and thus inhibited cell growth and infiltration by up-regulating PPAR-α (83). In addition, miR-29b expression was lower in placentas derived from patients with GDM than those in the control group (84). At a molecular level, hypoxia-inducible factor 3 alpha (HIF3A) was found to be a direct target of miR-29b, with two specific binding sites at the recognition sequences of miR-29b in the 3’-UTR of HIF3A mRNA, which, in turn, was negatively correlated with the miR-29b expression level (84). The other study regarding miR-29b drew a similar conclusion, suggesting that the effect of miR-29b was related to the PI3K/Akt signaling pathway at a molecular level (85). Moreover, miR-96 was down-regulated in GDM patients, which could enhance β-cell function, whereas serine/threonine-protein kinase (PAK)1 inhibited β-cell function and cell viability through functional analyses (86). Another study also showed that down-regulated miR-138-5p was associated with GDM, and significantly inhibited the migration and proliferation of trophoblasts (HTR-8/SVneo) by targeting the 3’-UTR of transducin (beta)-like 1 X-linked (88). Notably, a study performed by He et al. showed that down-regulated miR-494 had a protective role in pancreatic β-cell function by targeting the phosphatase and tensin homolog in GDM (95). Additionally, the study showed that down-regulation of miR-143 mediates the metabolic switch from oxidative phosphorylation to aerobic glycolysis in the placenta of women (89), highlighting its critical role in GDM. Furthermore, several studies also reported miRNAs, such as miR-155-5p (90), miR-20a-5p (82), and miR-181a (in umbilical-cord blood cells) (91), were down-regulated and related to GDM. These studies suggested that down-regulated miRNAs also had a profound influence in the pathogenesis of GDM.

In addition, besides studies based on samples from GDM patients, a few studies also highlighted the role of miRNAs in GDM *in vivo*. For example, Han et al. established a GDM rat model; based on this model, they found that miR-873 in GDM rats modulated insulin resistance and influenced myocardial apoptosis (96). With the downregulation of miR-873 in GDM rats, heart function was improved and myocardial apoptosis was inhibited (96). In addition, the expression of miRNA-122 was downregulated in the plasma of GDM rats, and miR-122 levels were regulated both *in vitro* through PPARγ activation and *in vivo* through a maternal diet enriched in PPAR ligands (87). Furthermore, the miRNA-221 level in the placental tissues of GDM rats was down-regulated compared with the control group; the overexpression of miRNA-221 stimulated insulin secretion, cell proliferation, and suppressed apoptosis *via* PAK1 in INS-1 cells (93). In addition, the up-regulation of miR-351 protected against insulin resistance and liver gluconeogenesis by repressing the PI3K/AKT pathway and regulating flotillin 2 in GDM mice; this highlights miR-351 as a potential therapeutic target for the clinical management of GDM (77). As for miR-142-3p, its upregulation promoted the survival of pancreatic β cells through targeting forkhead box protein O1 (FOXO1), suggesting that the targeted regulation of miR-142-3p/FOXO1 might be a new strategy for the treatment of GDM (71). The study performed by Tang et al. showed that miR-335-5p promoted insulin resistance and suppressed pancreatic islet β-cell secretion in GDM by activating the TGF-β signaling pathway and suppressing the expression of vasohibin-1 (74). Moreover, human embryonic stem cells were induced to express miR-410, which directly targeted lactate dehydrogenase A, a gene selectively repressed in normal insulin secreting β-cells, and thus improved glucose metabolism and reproductive outcome (98). These *in vivo* studies provide evidence that miRNAs are involved in the pathophysiology of GDM.

Additionally, several *in vitro* studies suggested the differential expression of miRNAs in GDM. For instance, Liu et al. performed bioinformatics analyses based on a gene expression dataset of fetoplacental arterial and venous endothelial cells from GDM and healthy control groups. They found that 11 miRNAs (including hsa-miR-1299, hsa-miR-1200, hsa-miR-578, hsa-miR-593-5p, hsa-miR-765, hsa-miR-520d-3p, hsa-miR-617, hsa-miR-92a-3p, hsa-miR-30b-5p, hsa-miR-181c-5p, and hsa-miR-181a-5p) differed in their expression (99), indicating their potential role in GDM; however, future studies are needed to elucidate this further. Additionally, miR-195-5p was up-regulated in GDM-induced HUVECs, inhibited cell viability and proliferation, and promoted apoptosis by targeting EZH2 *in vitro* (72), thus highlighting the importance of miRNAs in GDM.

### Role of circRNAs in GDM

The role of circRNAs in GDM remains largely unknown. Tang et al. reported that 55 circRNAs were up-regulated while 59 circRNAs were down-regulated in GDM placentas compared with those of healthy controls (41), highlighting the potential role of circRNAs in this disease. Gene Ontology (GO) analysis on genes of differentially expressed circRNAs showed the most significantly enriched term was activation of phospholipase C activity in biological processes. The most significantly enriched term was phosphatidylinositol 3-kinase complex in cellular component, and the most significantly enriched term was phosphatidylinositol binding in molecular function. Additionally, of the top 10 enrichment pathways in Kyoto Encyclopedia of Genes and Genomes (KEGG) analysis, the most enriched pathway was the Rap1 signaling pathway (41). Interestingly, the study by Wang et al. identified 46 circRNAs that were differentially expressed in the placenta, including three that were upregulated and 43 that were downregulated based on 30 GDM patients and 15 healthy controls (100). Further KEGG analysis showed they may be involved in advanced glycation end-products receptor for advanced glycation end-products signaling pathway in diabetic complications (100), which indicated that circRNAs might participate in the occurrence and pathogenesis of GDM. Furthermore, Yan et al. also performed RNA-seq to identify the circRNAs involved in GDM, with 48,270 circRNAs from the placental villi of GDM and control groups sequenced (101). Of these, 227 circRNAs were significantly up-regulated and 255 circRNAs were significantly down-regulated. Further GO and KEGG biological pathway analyses demonstrated that glycometabolism and lipometabolism processes, which are important in GDM development, were significantly enriched (101).

In addition, several studies reported that some specific circRNAs played a vital role in GDM (**Table 1**). For example, the expression of hsa_circ_0005243 was significantly reduced in both the placenta and plasma of GDM patients compared with healthy controls (55). At a molecular level, the knockdown of hsa_circ_0005243 significantly suppressed cell proliferation and migration, mainly by reducing the expression of β-catenin, and increased nuclear NF-κB p65 nuclear translocation *in vitro* (55). In addition, hsa_circ_0054633 was highly expressed in the blood and placenta during the second and third trimesters of pregnant GDM patients compared with healthy controls (54), suggesting its potential function in GDM. However, more research is required to gain an insight into the pathogenesis of GDM from the aspect of circRNAs, and to verify their clinical value.

### Role of EVs in GDM

During pregnancy, an increase occurs in the number of EVs, especially placental EVs in maternal plasma. Although the role of EVs during GDM remains to be fully elucidated, EVs profiles may be of diagnostic utility in screening asymptomatic populations (102). The previous study showed that the size of the EVs obtained in the first trimester of pregnancy was very similar between GDM patients and healthy controls; however, the concentration of EVs collected in the first trimester was significantly higher in GDM patients (103). As for the function of EVs, a previous study suggested that EVs extracted from the plasma of pregnant women with GDM significantly increased the release of inflammatory cytokines from endothelial cells (104), which might be related to the inflammatory process in GDM. In addition, HUVECs also released EVs *in vitro*; a related study found that EVs released from HUVECs from normal pregnancies could reverse a GDM phenotype, while EVs from HUVECs from GDM pregnancies carried factors that induced dysfunction in endothelial cells from normal pregnancies (105). Furthermore, although the mechanisms underpinning maternal metabolic adaptations to a healthy pregnancy and in GDM remain poorly understood, EVs may represent a novel mechanism regulating maternal glucose homeostasis in pregnancy. For example, James-Allen et al. found that small EVs isolated from healthy pregnant women promoted islet glucose-stimulated insulin secretion and peripheral insulin resistance in nonpregnant mice; EVs from GDM women failed to stimulate insulin secretion and caused exacerbated insulin resistance (106). These studies suggest altered sEV content contributes to the development of GDM.

EVs contain a variety of intracellular components and serve as important carriers for mediating intercellular communication (**Table 3**). Notably, lncRNAs might be one of main components of EVs. Based on the microarray technique, Cao et al. (107) identified 84 differentially expressed mRNAs and 256 differentially expressed lncRNAs in umbilical cord blood EVs of GDM patients compared with normal controls. Further bioinformatics analyses suggested that metabolic processes, growth, and development were significantly enriched through GO and KEGG analyses (107). However, the role of lncRNAs in EVs remains largely unknown, with further studies required.

**Table 3:** Extracellular vehicles and their cargos in GDM.

Cargo	Detection Methods	Key Points of Investigation	Model (*In Vitro, In Vivo*, Human)	Sample Type	Sample Size	Ref.
lncRNA, mRNA	Microarray	84 mRNAs and 256 lncRNAs differentially expressed in EVs of GDM patients compared with controls	Human	Umbilical cord blood	23 GDM vs 23 controls	(107)
miRNA	RT-qPCR	miR-222-3p, miR-516b-5p, miR-16-5p, miR-517-3p and miR-518-5p	Human	Urine	27 GDM vs 34 controls	(108)
miRNA	RT-qPCR	miR-122-5p; miR-132-3p; miR-1323; miR-136-5p; miR-182-3p; miR-210-3p; miR-29a-3p; miR-29b-3p; miR-342-3p, and miR-520h	Human	Peripheral blood	23 GDM vs 46 controls	(109)
miRNA	Next generation sequencing	Differentially expressed miRNAs in EVs unveil that they target genes associated with glucose homeostasis and metabolism.	Human	Chorionic villi explant culture, skeletal muscle tissue and plasma samples	12 GDM vs 12 controls	(110)
circRNA	Microarray	229 circRNAs were significantly up-regulated and 278 circRNAs were significantly down-regulated in the GDM patients	Human	Umbilical cord blood	23 GDM vs 23 controls	(111)
Proteins	Mass spectrometry	Ingenuity pathway analysis of the exosomal proteins revealed differential expression of the proteins targeting the sirtuin signaling pathway, oxidative phosphorylation, and mechanistic target of rapamycin signaling pathway	Human	Adipose tissue	82 GDM vs 65 controls	(112)
Proteins	Mass spectrometry	Bioinformatic analysis shows that the exosomal proteins in GDM target pathways are mainly associated with energy production, inflammation, and metabolism	Human	Peripheral blood	11 GDM vs 11 controls	(113)
Proteins	Mass spectrometry	S100 calcium binding protein A9, damage associated molecular pattern signal, was found to be significantly increased in GDM	Human	Urine	8 GDM vs 10 controls	(114)

lncRNA, long non-coding RNA; miRNA, microRNA; GDM, Gestational diabetes mellitus.

The expression of miRNAs contained in placental EVs purified using different types of tissues from GDM patients has been evaluated. Placental EVs were purified from urine and the expression of five miRNAs (miR-516-5p, miR-517-3p, miR-518-5p, miR-222-3p, and miR-16-5p) was found to be downregulated in patients with GDM at the third trimester of gestation compared with healthy controls (108). As for peripheral blood, 10 miRNAs (miR-122-5p, miR-132-3p, miR-1323, miR-136-5p, miR-182-3p, miR-210-3p, miR-29a-3p, miR-29b-3p, miR-342-3p, and miR-520h) showed significantly higher levels in GDM cases than in healthy controls. Such miRNAs were related to trophoblast proliferation/differentiation as well as to insulin secretion/regulation and glucose transport through further bioinformatics analyses  (109). In addition, Nair et al. reported that GDM modified miRNA content in the EVs of chorionic villi explants when compared to those of healthy controls (110). Bioinformatic analysis of differentially expressed miRNAs revealed that they targeted genes associated with glucose homeostasis and metabolism (110). Moreover, five candidate miRNAs (miR-125a-3p, miR-99b-5p, miR-197-3p, miR-22-3p, and miR-224-386-5p) were significantly over-expressed in EVs released by GDM chorionic villi and were also upregulated in skeletal muscle tissue from GDM pregnancies compared to controls (110). Interestingly, placental EVs from healthy controls showed increased migration and glucose uptake in response to insulin in skeletal muscle from diabetics, suggesting placental EVs might have a role in changes of insulin sensitivity (110). These studies suggested that EVs might have a function in GDM *via* miRNAs, thus providing a new theoretical basis for research in the clinic.

Except for lncRNAs and miRNAs, circRNA was also found in EVs; these were discovered to carry signaling molecules for cellular communication and even organ crosstalk. A recent study has shown different expression patterns of exosomal circRNAs between GDM patients and healthy pregnant women (111). They identified 507 differentially expressed circRNAs in GDM patients compared with controls, and reported that several pathways were significantly enriched based on GO and KEGG analyses, including the pentose phosphate pathway, cholesterol metabolism, galactose metabolism, as well as other pathways (111). Therefore, the roles of circRNAs in EVs are still limited and needs to be further verified by animal and clinical studies.

The proteins contained in EVs might also differ in GDM patients. Jayabalan et al. showed that the number of EVs from adipose tissue was substantially (1.7-fold) greater in GDM than that in healthy controls (112). Ingenuity pathway analysis of exosomal proteins revealed the differential expression of proteins targeting the sirtuin signaling pathway, oxidative phosphorylation, and mTOR signaling pathway in GDM compared with controls (112), highlighting how EVs from adipose tissue played an important role in mediating changes in placental function in GDM. As for peripheral blood, one study reported a total of 78 statistically significant proteins in the relative expression of exosomal proteins in GDM compared with healthy controls (113). Bioinformatic analysis showed that the exosomal proteins in GDM target pathways were mainly associated with energy production, inflammation, and metabolism (113). In addition, the EVs proteome content from urine samples of pregnant patients with GDM was compared with that of controls, identifying 646 and 734 proteins in EVs from urine samples of controls and GDM patients, respectively (114). Notably, S100 calcium binding protein A9, a damage-associated molecular pattern signal, was found to be significantly increased in GDM (114), providing insights into maternal changes during diabetic pregnancy. Further research to explore the role of types of proteins in EVs and clinical tests to confirm their importance are needed in the future.

## Clinical Significance of Non-Coding RNAs and EVs for GDM

### Non-coding RNAs and EVs as Early Diagnostic Biomarkers

Early diagnosis is essential in reducing GDM-associated complications, for both the mother and fetus, by implementing treatments that normalize blood glucose levels. However, the current common approach in screening method based on plasma glucose measurements identifies women at late stages of GDM (24–28 weeks of gestation) (115). This means that treatment cannot start until 24–28 weeks of gestation, which already presents a high risk of fetal morbidity and mortality. Additionally, effective early identification of the development of GDM might also reduce disease onset. Consequently, many researchers are investigating biomarkers potentially present in the blood to accurately diagnose GDM earlier than 24–28 weeks. Non-coding RNAs, especially miRNAs, show great potential as early-trimester biomarkers for GDM as they are highly stable in body fluids and are accessible from maternal fluids during early gestation. Recently, numerous studies have explored the potential role of non-coding RNAs as early diagnostic biomarkers in GDM. However, multi-center and large-scale clinical studies are currently lacking, and further clinical studies are needed to identify the value of non-coding RNAs and EVs as early diagnostic biomarkers for GDM in clinic.

Most studies have evaluated miRNAs as early diagnostic biomarkers for GDM (**Table 4**). Extracellular miRNAs exist in blood that could be quantified rapidly in a clinical setting. Such a property makes it possible to use miRNAs as biomarkers for the diagnosis of GDM in the clinic. For example, Zhu et al. (64) conducted a prospective pilot study by collecting peripheral blood samples from pregnant women at 16−19 weeks in 2015 and identified 32 differentially expressed miRNAs in GDM using high−throughput sequencing. The differential expression of five upregulated miRNAs, including miR-16-5p, miR-17-5p, miR-19a-3p, miR-19b-3p, and miR-20a-5p, was confirmed by quantitative reverse transcription–PCR. Therefore, these miRNAs were thought to predict GDM at an early stage of pregnancy. All five miRNAs were further investigated by Cao et al. (117). In this study, researchers collected plasma from 157 pregnant participants at the first prenatal examination and then every 4 weeks until testing for GDM, and the expression of miRNAs was then analyzed. They also reported that the relative and absolute expression of plasma miRNA-16-5p, miR-17-5p, and miR-20a-5p from GDM women were significantly up-regulated compared with controls, with areas under the curve (AUC) of 0.92 (95% CI: 0.871–0.984), 0.88 (95% CI: 0.798–0.962), and 0.74 (95% CI: 0.618–0.870), respectively (117). Another study performed by Wander et al. (61) measured early to mid-pregnancy plasma levels of 10 miRNAs in GDM and found that miR−21−3p and miR−210−3p were associated with a higher risk of GDM in women who were overweight/obese prior to pregnancy, while several miRNAs, including miR−155−5p, miR−21−3p, miR−146b−5p, miR−223−3p, miR−517−5p, and miR−29−3p, were associated with an increased risk of GDM in mothers bearing male offspring. Furthermore, Yoffe et al. (118) evaluated the potency of first trimester serum miRNAs as new early diagnostic biomarkers for GDM patients in two countries (Italy and Spain), and reported that miR-223 and miR-23a were significantly increased in GDM women compared to healthy pregnant women. As for miR-223 and miR-23a, the former was a slightly better classifier than miR-223+miR-23a or miR-23a alone: AUC = 0.94 and accuracy = 0.90 for miR-223; AUC = 0.89 and accuracy = 0.90 for miR-23a; and AUC = 0.91 and accuracy= 0.90 for miR-223+miR-23a (118). In addition to up-regulated miRNAs in GDM, Zhao et al. (62) found miR-132, miR-29a, and miR-222 in serum collected between 16 and 19 gestational weeks were significantly decreased in GDM women with respect to controls.

**Table 4:** Selected ncRNAs as biomarkers for GDM.

Type of ncRNA	Expression in GDM	ROC area under curve	Sample Size	Ref.
		(95%CI)	
lncRNA MEG8	Up	0.73 (0.67-0.78)	78 GDMs vs 322 controls	(116)
miR-16-5p	Up	0.92 (0.87-0.98)	85 GDM vs 72 controls	(117)
miR-17-5p	Up	0.88 (0.80-0.96)	85 GDM vs 72 controls	(117)
miR-20a-5p	Up	0.74 (0.62-0.87)	85 GDM vs 72 controls	(117)
miR-23a	Up	AUC = 0.89 and accuracy = 0.90	23GDM vs 20 controls	(118)
		for miR-23a		
miR‐195‐5p	Up	0.85 (0.79-0.90)	102 GDM vs 102 controls	(119)
miR-223	Up	AUC = 0.94 and accuracy = 0.90	23GDM vs 20 controls	(118)
		for miR-223		
miR-330-3p	Up	–	31 GDM vs 29 controls	(120)
miR-21-3p	Down	AUC= 0.73	19 GDM vs 28 controls	(121)
miR-29a/b	Down	0.83 (0.76-0.90) for miR-29a,	68 GDM vs 55 controls	(122)
		0.86 (0.79-0.93) for miR-29b		
miR-185	Down	AUC= 0.93	156 GDM vs 100 controls	(123)
miR-132	Down	AUC= 0.90	108 GDM vs 50 controls	(124)
Hsa_circRNA_0054633	Up	0.79 (0.69-0.90) (2nd trimester)	40 GDM vs 40 controls (2nd trimester), 65 GDM vs 65 controls (3rd trimester)	(54)
Hsa_circRNA_102893	Down	0.81 (0.59–0.94) for training set,	12 GDM vs 12 controls (training set),	(56)
		0.74 (0.57–0.87) for test set	18 GDM vs 18 controls (test set)	
exosomal miR-16-5p	Up (2nd trimester), Down (3rd trimester)	1.00 (1.00-1.00) (2nd trimester)	27 GDM vs 34 controls	(108)
exosomal miR-222-3p	NS (2nd trimester), Down (3rd trimester)	0.69 (0.29-1.09) (2nd trimester)	27 GDM vs 34 controls	(108)
exosomal miR-516b-5p	NS (2nd trimester), Down (3rd trimester)	0.94 (0.76-1.11) (2nd trimester)	27 GDM vs 34 controls	(108)
exosomal miR-517-5p	Up (2nd trimester), Down (3rd trimester)	1.00 (1.00-1.00) (2nd trimester)	27 GDM vs 34 controls	(108)
exosomal miR-518-3p	Up (2nd trimester), Down (3rd trimester)	1.00 (1.00-1.00) (2nd trimester)	27 GDM vs 34 controls	(108)
Oral extracellular vesicles	Up	AUC= 0.81	11 GDM vs 23 controls	(125)

AUC, area under curve; circRNA, circular RNA; lncRNA, long non-coding RNA; miRNA, microRNA; GDM, Gestational diabetes mellitus; ROC, Receiver operating characteristic curve.

The differential expression of other miRNAs in GDM and normal pregnancies was also identified in the later stages of pregnancy rather than before 24–28 weeks’ gestation. For example, Zhou et al. found a receiver operating characteristic curve (ROC) showed that serum miR-132 (24-28 weeks) had considerable diagnostic accuracy with an AUC of 0.898 for GDM (124). MicroRNA-195-5p expression was significantly increased in serum samples from GDM patients as compared with that in healthy pregnancies with an AUC of 0.8451 (119). Another study through ROC analysis showed that the AUC was 0.927 for miR-185 with a sensitivity and specificity of 0.865 and 0.838, respectively, indicating serum miR-185 differentiated patients with GDM from healthy controls (123). Moreover, miR-330-3p (18, 24–32) was identified as being significantly upregulated in lean women with GDM compared to nondiabetic pregnant women during the third trimester, highlighting miR-330-3p as a possible new biomarker (120). Furthermore, a significant decrease occurred in the expression levels of miR-21-3p during the third trimester in GDM patients compared with healthy controls, whose AUC in GDM was 0.73 (121). Serum miR-29a and miRNA-29b were also down-regulated in GDM patients compared with controls; the AUC was 0.829 for the diagnosis of GDM using serum miR-29a expression, 0.857 for a diagnosis using serum miR-29b expression, and 0.944 for a combined diagnosis (using both miR-29a and miR-29b) (122). These new miRNAs may be novel diagnostic circulating biomarkers and have the potential to be developed into new interventional targets for patients with GDM. Therefore, miRNAs could play a critical role in diagnosis and predictive capability or even as treatment targets for GDM in the clinic.

Except for miRNAs, only a few studies have explored the role of lncRNAs, circRNAs, and EVs as early diagnostic biomarkers in GDM (**Table 4**). For example, lncRNA MEG8 might be a biomarker for the early diagnosis of GDM. A study conducted by Zhang et al. suggested that patients with a high pre-pregnancy plasma level of lncRNA MEG8 showed a high incidence rate of GDM during pregnancy, with plasma levels of lncRNA MEG8 one month before a diagnosis of GDM sufficient to distinguish GDM patients from healthy controls (116). Notably, circRNAs also have the potency to become new biomarkers for GDM. For instance, Yang et al. reported that the circulating expression of hsa_circRNA_102893 was down-regulated and contributed to the early detection of GDM, whose areas under ROC were 0.806 (95% CI 0.594–0.937) and 0.741 (0.568–0.872) in training and test sets, respectively (56). Additionally, the AUC of hsa_circRNA_0054633 during the second trimester was 0.793 (0.685–0.901) and its best sensitivity and specificity were 57.6% and 90.9%, respectively (54).

As for EVs, EVs might also become new early diagnostic biomarkers for GDM. Total EVs isolated from gingival crevicular fluid were significantly higher in patients who developed GDM later in pregnancy compared to normoglycemic pregnant women; the concentration of extracellular vesicles delivered an area under the ROC curve of 0.81 (125). The miRNAs from EVs are suggested to be new biomarkers for the early diagnosis of GDM. The study revealed that the AUC was 1 (95% CI 1-1) for exosomal miR-16-5p, miR-517-3p, and miR-518-5p in the early second trimester, indicating the high diagnostic accuracy of these miRNAs for differentiating between patients with GDM and healthy women (108). These studies showed that non-coding RNAs and EVs could be considered as good early markers for the diagnosis of GDM. However, several limitations regarding non-coding RNAs and EVs as biomarkers for GDM should be acknowledged. First, as mentioned previously, more multi-center and large-scale clinical studies are warranted to evaluate the potential role of each biomarker for GDM. Additionally, whether each biomarker has the same or similar different expression trend in population of different races is still unclear and need further studies to clarify. What’s more, in order to increase the diagnostic accuracy of GDM, which biomarkers could be used in combination are unknown and need to be verified in future studies. Finally, as we know, environmental factors such as diet and physical activity play important roles in GDM, therefore, whether and how environmental factors have impact on diversity and expression level of biomarkers need to be figured out.

### Non-Coding RNAs as Predictive Biomarkers in the Development of T2DM and CVDs in Mothers and Offspring

Women with GDM have an increased risk of developing diabetes (predominantly T2DM) and cardiovascular diseases (CVD) later in life. It is estimated that up to 70% of women with GDM will develop diabetes within 22–28 years after pregnancy (126–128). Early identification of a high-risk group of mothers at risk of later development of T2DM and CVD can lead to early primary prevention strategies and long-term follow-up, reducing disease onset. Several miRNAs, as stable and accessible biomarkers in peripheral blood, have been investigated for a potential role as early predictive biomarkers in the development of T2DM and CVD in mothers with a history of GDM. Hromadnikova et al. (129) explored the expression profiling of miRNAs associated with diabetes mellitus and CVDs in whole peripheral blood of young and middle-aged mothers with a history of GDM 3–11 years after delivery. They found that 16 miRNAs (miR-1-3p, miR-16-5p, miR-17-5p, miR-20b-5p, miR-21-5p, miR-23a-3p, miR-26a-5p, miR-29a-3p, miR-103a-3p, miR-133a-3p, miR-146a-5p, miR-181a-5p, miR-195-5p, miR-199a-5p, miR-221-3p, and miR-499a-5p) with an aberrant postpartum expression profile in the whole peripheral blood of mothers with a prior exposure to GDM showed higher sensitivity, ranging from 15.32% to 43.24%, at a 10.0% false positive rate (FPR) in predicting the development of T2DM and CVDs (129). Further analysis demonstrated that screening based on a combination of these particular miRNAs was superior to using individual miRNAs since it showed the highest accuracy for mothers with a history of GDM (AUC 0.900, *p* < 0.001, sensitivity 77.48%, specificity 93.26%) (129). Screening was able to identify 77.48% of mothers with an increased cardiovascular risk at 10.0% FPR. Therefore, screening of particular miRNAs may stratify a high-risk group of mothers with a history of GDM that might benefit from the implementation of early primary prevention strategies, follow by a reduction in the onset of later T2DM and CVDs.

In addition, it is believed that fetal exposure to a hostile environment in GDM pregnancies might induce modifications in the non-coding RNA expression profile, therefore promoting changes in gene expression in offspring and affecting children in the development of diabetes mellitus and CVDs. Hromadnikova et al. (130) collected whole peripheral venous blood from children aged 3 to 11 years prenatally exposed to GDM and assessed the expression profile of miRNAs associated with diabetes and CVDs. The goal was to assess to what extent fetal and environmental programming predispositions had a significant impact on the risk of developing diabetes and CVDs in adulthood. They demonstrated that several miRNAs associated with diabetes and CVDs, including miR-1-3p, miR-17-5p, miR-20a-5p, miR-20b-5p, miR-21-5p, miR-29a-3p, miR-103a-3p, miR-126-3p, miR-133a-3p, miR-143-3p, miR-181a-5p, miR-195-5p, miR-210-3p, miR-221-3p, and miR-499a-5p, were dysregulated in children from GDM complicated pregnancies regardless of the occurrence of postnatal clinical findings (130). In addition, seven miRNAs (miR-16-5p, miR-23a-3p, miR-26a-5p, miR-100-5p, miR-125b-5p, miR-146a-5p, and miR-574-3p) were dysregulated in children with a prior exposure to GDM and with normal postnatal clinical findings. Only the differential expressions of miR-92a-3p and miR-155-5p were observed in children with affected GDM, who were found to have abnormal clinical findings. Likewise, screening based on a combination of miRNAs was superior over using individual miRNAs in the assessment of the potential risk of children in the later development of diabetes mellitus and CVDs. At 10.0% FPR, screening was able to identify 75.41% of children with normal clinical findings and 96.49% of children with abnormal clinical findings using an aberrant miRNA expression profile (130). Therefore, aberrant miRNAs also had a vital role in predicting the development of T2DM and CVDs in offspring affected by GDM. Further studies with a large size sample are needed to verify miRNAs and explore other non-coding RNAs, including lncRNAs and circRNAs, as early biomarkers in predicting the development of T2DM and CVDs in mothers and offspring.

### Non-Coding RNAs and EVs as Therapeutic Targets in GDM

According to American Diabetes Association guidelines, the main therapies for GDM are a lifestyle improvement and insulin injections. However, insulin therapy did not lead to mothers with severe hyperglycemia achieving a target of glycemic control because of insulin resistance pathophysiological features in GDM. Recently, a few studies have explored therapeutic targets of GDM based on its etiology. As mentioned previously, both non-coding RNAs and EVs play vital roles in the pathogenesis of GDM based on clinical and experimental research, and thus are attractive therapeutic targets in GDM. For example, the study performed by Mi et al. (98) transduced miR-410 into human embryonic stem cells (hESCs) using a Tet-on system. These miR-410-transduced hESCs were further differentiated into pancreatic endoderm (PE) cells and transplanted into db+/+ female mice (98). The results demonstrated that the miR-PE transplant alleviated hyperglycemia and hyperinsulinemia in pregnant female mice, and significantly improved their reproductive outcome. In addition, James-Allan et al. (106) developed a novel mouse model involving jugular vein catheterization and mini-osmotic pumps to mimic physiological conditions by chronically infusing sEVs from nonpregnant women, healthy pregnant women, and women diagnosed with GDM into healthy nonpregnant mice. The study showed that animals infused with sEVs from GDM developed glucose intolerance. Also, glucose-stimulated insulin secretion was increased in mice infused with healthy pregnancy sEVs compared to mice receiving nonpregnant sEVs. Therefore, sEVs hold promise as a novel therapeutic approach for pathologies associated with GDM. Considering the safety of therapy in GDM, research focusing on therapeutic targets in GDM are limited.

## Conclusion and Further Prospects

RNA sequencing has provided an unprecedented insight into the human genome. The diversity of responses that are observed in different tissues and diseases demonstrates the complex functions of non-coding RNAs in the body. The field of non-coding RNAs is growing at a blistering pace with several clinics and labs investigating various diseases, including GDM. This review will help to provide a better understanding of lncRNAs, miRNAs, and circRNAs in the pathogenesis of GDM. The studies described herein provide strong evidence that non-coding RNAs play important roles in GDM, but a number of important questions remain unanswered. Notably, an abundance of studies have explored the role of miRNAs in the pathogenesis of GDM; however, research on lncRNAs and circRNAs in GDM is far from complete. The further studies should focus on non-coding RNAs that are obviously different expression in GDM, and identify their potential function in GDM through bioinformatic analyses. The bioinformatic analyses could give the hints regarding target genes and specific biological processes regulated by non-coding RNAs, which is helpful for conducting experiments. Besides, more thorough studies explore functional characterization of specific non-coding RNAs in GDM are needed, especially regarding the roles of lncRNAs in GDM. Considering lncRNAs could exert their functions by directly binding to DNA, RNA, and proteins participating in the transcriptional and post-transcriptional regulation, future studies should focus on specific lncRNAs and explore how they influence GDM, which may provide novel insights into the molecular mechanisms governing GDM. In addition, other limitations are associated with using circulating or tissue specific non-coding RNAs as biomarkers. What is more, the therapeutics value of non-coding RNAs needs to be evaluated in order to develop new targets for clinical use.

As for EVs, a few studies have highlighted the important role of these in GDM by illustrating the potential mechanisms of GDM from another aspect. Clinical studies of EVs, including their use as drug-delivery systems, have shown promising results, highlighting these as potential therapeutic targets for GDM. Gaining a better understanding of the roles of EVs in GDM will provide novel insights into the molecular mechanisms governing GDM. However, the clinical value and identification of more exact molecular mechanisms regarding EVs in GDM are still needed, which will help reveal the diagnostic and therapeutic potential of EVs in GDM.

Therefore, future work on the involvement of non-coding RNA and EVs in GDM might include the following:

1) A thorough functional characterization of specific non-coding RNAs in GDM, at both molecular and cellular levels;
2) Retrieving the most promising non-coding RNA candidates for therapeutic targets from the huge amount of sequencing data available;
3) Identifying panels of specific non-coding RNAs for their optimal accuracy in the early diagnosis of GDM;
4) Evaluating non-coding RNA patterns from a very early stage of pregnancy and then during different windows of time until delivery to ascertain the reliability of non-coding RNAs as independent predictors of GDM;
5) Carrying out more research on lncRNA-based or circRNA-based therapy *in vivo* using optimal delivery systems;
6) Effect of crosstalk of EV-derived cargo among different tissues in GDM;
7) Evaluating the therapeutic possibility of using EVs *in vivo* in GDM.

## Author Contributions

T-NZ, WW, X-MH, and S-YG wrote the review. All authors contributed to the article and approved the submitted version.

## Funding

This work was supported by China Postdoctoral Science Foundation funded project (No. 2019M661178 to S-YG), 345 Talent Project of Shengjing Hospital of China Medical University (No. M0334 to S-YG and No. M0691 to T-NZ).

## Conflict of Interest

The authors declare that the research was conducted in the absence of any commercial or financial relationships that could be construed as a potential conflict of interest.
